# A patient’s perception of their hospital stay influences the functional outcome and satisfaction of total knee arthroplasty

**DOI:** 10.1007/s00402-017-2661-7

**Published:** 2017-03-22

**Authors:** N. D. Clement, D. Macdonald, R. Burnett, A. H. R. W. Simpson, C. R. Howie

**Affiliations:** 1 Department of Orthopaedics and Trauma, The Royal Infirmary of Edinburgh, Little France, Edinburgh, EH16 4SA UK; 2 University of Edinburgh, Little France, Edinburgh, EH16 4SB UK

**Keywords:** Total knee arthroplasty, Hospital, Stay, Experience, Satisfaction, Outcome

## Abstract

**Introduction:**

To assess whether patient satisfaction with their hospital stay influences the early outcome of total knee arthroplasty (TKA).

**Methods:**

During a 5-year period patients undergoing primary TKA at the study centre had prospective outcome data recorded (*n* = 2264). The Oxford knee score (OKS) and the short form (SF)-12 were recorded pre-operatively and 1 year post-operatively when satisfaction with their TKA was also assessed. Patient satisfaction with their hospital stay was also evaluated and their reasons for it were qualitatively documented.

**Results:**

Decreasing level of satisfaction with their hospital stay was associated with a significantly worse post-operative OKS (*p* < 0.001) and SF-12 score (*p* < 0.001). Multivariable regression analysis confirmed that the patient’s perceived level of satisfaction with their hospital stay was an independent predictor of change in the OKS (*p* < 0.001) and SF-12 score (*p* < 0.006) after adjusting for confounding variables. Patient satisfaction with their TKA was significantly influenced by their hospital experience, decreasing from 96% in those with an excellent experience to 42% in those with a poor experience. Food, staff/care, and the hospital environment were the most frequent reasons of why patients rated their hospital experience as fair or poor.

**Conclusion:**

A patient’s perception of their inpatient hospital experience after surgery is an important modifiable predictor of early functional outcome and satisfaction with TKA.

## Introduction

The outcome of total knee arthroplasty (TKA) according to patient reported outcome measures (PROMs) is variable and dependent upon multiple factors [1]. Such PROMs have been demonstrated to correlate with patient satisfaction 1 year following TKA [15, 21]. However, the rate of patient satisfaction after TKA varies from 75 to 92% [13, 20]. Pre-operative mental health and improvement in both generic health scores and joint specific scores have been shown to be independent predictors of patient satisfaction after TKA [13, 21]. Whether patients’ subjective experience of their hospital care after a TKA effects their satisfaction with the TKA has not previously been evaluated.

Baumann et al. [3] demonstrated that patients satisfied with the quality of their hospital stay had significantly greater Short Form (SF-) 36 scores 1 year after TKA. The SF-36 is a generic health questionnaire, and the effect upon a joint specific questionnaire may not be equivalent. Furthermore, they did not assess patient satisfaction with their TKA, which may not be affected by the patient’s general health status. The Oxford knee score (OKS) is a widely used and accepted joint specific score [11], and has been shown to correlate with patient satisfaction after TKA [15]. If patient perceived satisfaction with their hospital care influences their functional outcome and satisfaction with their TKA, by improving the quality of their hospital stay post-operatively may result in a superior outcome.

The primary aim of this study was to assess whether patient satisfaction with hospital stay influences the early functional outcome of TKA, measured by both generic and joint specific PROMs, and if it effects patient satisfaction with their TKA. The secondary aim was to identify pre-operative predictors of satisfaction with hospital stay after a TKA. The null hypothesis was that satisfaction with hospital stay does not influence the early functional outcome of TKA and that satisfaction with stay cannot be predicted.

## Patients and methods

Prospective functional outcome data was recorded during a 5 year period (2006 to 2010) for patients undergoing TKA at the study centre. Patient demographics and comorbidities were recorded at the pre-operative assessment. Categories of comorbidity included were: heart disease, hypertension, lung disease, vascular disease, neurological problems, stomach ulcer, kidney disease, liver disease, depression, and concomitant back pain, which were recorded as dichotomous variables. OKS [11] and the SF-12 scores [22] were recorded pre-operatively and at 1 year post-operatively. The OKS consists of 12 questions assessed on a Likert scale with values from 0 to 4, a summative score is then calculated where 48 is the best possible score (least symptomatic) and 0 is the worst possible score (most symptomatic).

Patient satisfaction with the hospital stay during their TKA was assessed at 6 months review by asking the question: “How satisfied were you with your hospital experience?”, which was measured using a five point Likert scale: excellent, very good, good, fair, and poor. Patients were asked to record in a free text box what the worst aspect of their hospital stay was. To evaluate the reasons why their hospital experience was impaired the responses were categorised into issues associated with: food, staff, environment, pain, complications, multiple, and other to allow categorical analysis. A word cloud was also generated using WordCloud [19], the size of the words in the cloud reflect the frequency of use with a larger word signifying more frequent use.

Patient satisfaction with their TKA was assessed by asking the question “How satisfied are you with your operated knee?” 1 year after surgery. The response was recorded using a four point Likert scale: very satisfied, satisfied, neutral, and unsatisfied. Patients who recorded very satisfied or satisfied were classified as satisfied, which has been used previously to assess patient satisfaction after TKA [6].

During the study period the most commonly performed TKAs were the Kinemax (*n* = 258, Stryker Howmedica Osteonics, Allendale, New Jersey), Triathlon (*n* = 1233, Stryker), and the PFC Sigma (*n* = 773, DePuy, Johnson & Johnson Professional Inc, Raynham, Massachusetts). The majority of prostheses were cruciate retaining or deep dish cruciate substituting (*n* = 2219, 98%). All patients were reviewed at a pre-assessment clinic. A standardised rehabilitation protocol was used for all patients, with active mobilisation on the first day post-operatively. Length of stay was recorded. Patients were then reviewed at 6 weeks, 6 and 12 months post-operatively.

### Statistical analysis

Statistical analysis was performed using Statistical Package for Social Sciences version 17.0 (SPSS Inc., Chicago, IL, USA). Patients were categorised into groups according to their perceived level of satisfaction with their hospital stay (excellent, very good, good, fair, and poor). A Student’s *t* test, unpaired and paired, and a one way analysis of variance (ANOVA) were used to compare linear variables between groups. Post hoc analysis (with Bonferonni correction) was used to demonstrate between which groups there were significant differences identified on one way ANOVA. Dichotomous variables were assessed using a Chi square test. Logistic regression analysis was used to identify independent pre-operative predictors of patient perceived good to excellent satisfaction with their hospital stay, and to identify the independent effect of satisfaction with hospital stay on patient satisfaction with their TKA at 1 year. Multivariable linear regression analysis was used to assess the independent effect of satisfaction of hospital stay on change in the OKS, SF-12 physical component summary (PCS) and mental component summary (MCS) scores 1 year after TKA. All variables were included in all regression models using enter methodology. Multi-collinearity analysis prior regression analysis and collinear variables were identified and those with the lowest tolerance were removed, to produce a stable model with a variance inflation factor of <2. A *p*-value of less than 0.05 was defined as significant.

Ethical approval was obtained for analysis and publication of the presented data from the regional ethics committee.

## Results

There were 2392 TKA performed during the study period, however 128 patients did not record their level of satisfaction with their hospital stay and were excluded from analysis. The study cohort consisted of 2264 patients, of which 963 (42.5%) were male and 1301 (57.5%) females, with a mean age of 70.3 (SD 8.8, range 33–91) years and 70.5 (9.6, range 33–93) years, respectively. 876 (38.7%) patients did not have a medical comorbidity, with a median of one comorbidity (range 0–11). The most prevalent comorbidity was hypertension, affecting more than a third of patients (Table 1).

**Table 1:** Patient demographics and pre-operative functional scores according to their level of satisfaction with hospital stay

Demographic	Descriptive	Cohort (*n* = 2264)	Level of satisfaction with hospital stay				
			Excellent (*n* = 708)	Very good (*n* = 854)	Good (*n* = 429)	Fair (*n* = 184)	Poor (*n* = 89)
Gender (*n*, % group)	Male	963 (36.1)	341 (48.2)	355 (41.6)	153 (35.7)	75 (40.8)	39 (43.8)
	Female	1301 (48.8)	367 (51.8)	499 (58.4)	276 (64.3)	109 (59.2)	50 (56.2)
Age (years: mean, SD)		70.4 (9.3)	70.3 (9.1)	70.6 (9.4)	71.0 (9.3)	69.8 (9.8)	68.2 (8.50)
Comorbidity (*n*, % of group)	Heart disease	357 (13.4)	102 (14.4)	121 (14.2)	81 (18.9)	31 (16.8)	22 (24.7)
	Hypertension	956 (35.9)	295 (41.7)	342 (40.0)	190 (44.3)	88 (47.8)	41 (46.1)
	Lung disease	225 (8.4)	78 (11.0)	69 (8.1)	44 (10.2)	25 (13.6)	9 (10.1)
	Vascular disease	109 (4.1)	27 (3.8)	36 (4.2)	29 (6.8)	12 (6.5)	5 (5.6)
	Neurological disease	100 (3.8)	28 (4.0)	32 (3.7)	28 (6.5)	11 (6.0)	1 (1.1)
	Diabetes mellitus	265 (9.9)	86 (12.1)	93 (10.9)	47 (11.0)	25 (13.6)	14 (15.7)
	Gastric ulceration	96 (3.6)	35 (4.9)	30 (3.5)	19 (4.4)	11 (6.0)	1 (1.1)
	Kidney disease	54 (2.0)	17 (2.4)	12 (1.4)	14 (3.3)	8 (4.3)	3 (3.4)
	Liver disease	37 (1.4)	10 (1.4)	11 (1.3)	14 (3.3)	1 (0.5)	1 (1.1)
	Anaemia	135 (5.1)	39 (5.5)	44 (5.2)	40 (9.3)	10 (5.4)	2 (2.2)
	Back pain	792 (29.7)	215 (30.4)	291 (34.1)	167 (38.9)	84 (45.7)	35 (39.3)
	Depression	237 (8.9)	48 (6.8)	84 (9.8)	57 (13.3)	34 (18.5)	14 (15.7)
Length of stay (days: mean, SD)		6.0 (2.9)	5.8 (2.8)	6.0 (3.0)	6.2 (2.9)	6.2 (3.1)	6.2 (3.0)
Prosthesis (*n*, % of group)	PFC	773 (34.1)	234 (33.1)	297 (34.8)	148 (34.5)	68 (37.0)	26 (29.2)
	Triathlon	1233 (54.5)	387 (54.7)	458 (53.6)	233 (54.3)	96 (52.2)	59 (66.3)
	Kinemax	258 (11.4)	87 (12.3)	99 (11.6)	48 (11.2)	20 (10.9)	4 (4.5)
Functional measures							
OKS	Pre-operative (SD)	18.9 (7.5)	19.3 (8.1)	19.4 (7.2)	18.4 (7.4)	17.5 (7.0)	16.9 (7.1)
SF-12 PCS	Pre-operative (SD)	29.4 (7.2)	29.9 (7.9)	29.6 (7.0)	28.7 (6.6)	28.6 (6.8)	29.5 (7.6)
SF-12 MCS	Pre-operative (SD)	47.7 (12.0)	50.0 (11.5)	48.5 (11.5)	45.4 (11.7)	44.1 (12.9)	41.0 (13.8)

**Fig. 1:**
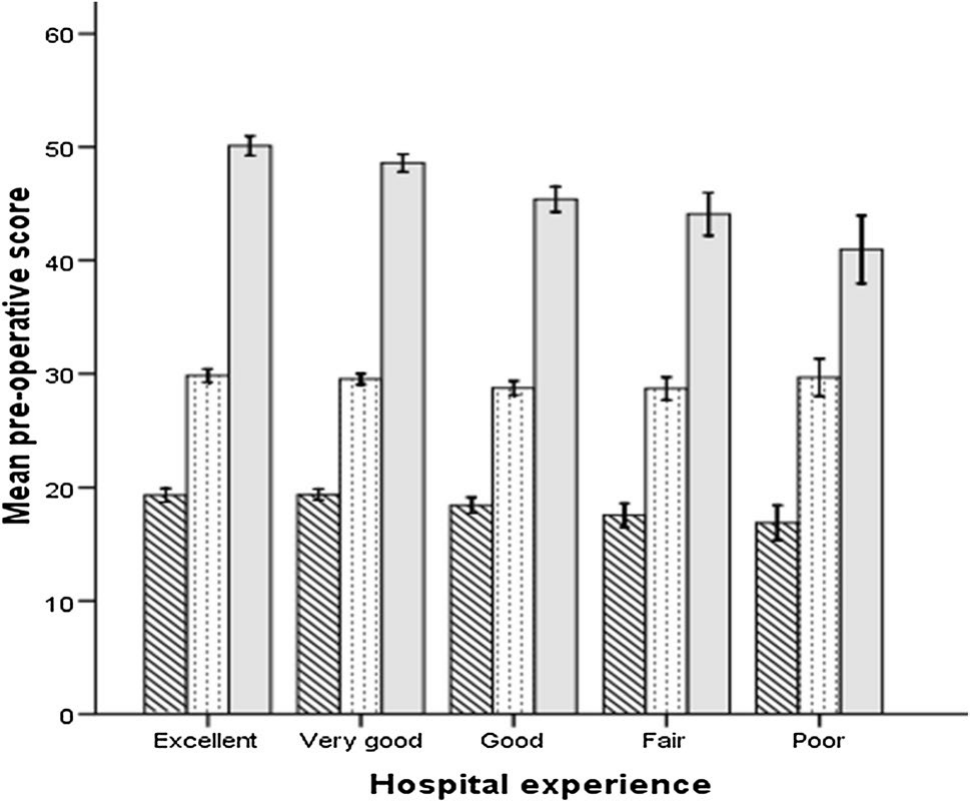
Pre-operative OKS (*diagonal lines*), SF-12 PCS (*dots*) and MCS (*grey*) according to level of patient satisfaction with their hospital experience. 95% confidence interval *error bars*

There were 1991 patients (88%) who rated their hospital stay as good to excellent, with 273 patients (12%) declaring that their hospital stay was either fair or poor. Female gender was associated with an increased risk of lower level of satisfaction with hospital stay (Table 1). There was no difference in age between groups, but there was a trend towards decreased satisfaction with younger age. Patients with heart disease, concomitant back pain, and or depression were more likely to have a decreased level of satisfaction with their hospital stay (Table 1). There was no difference in the length of stay between the groups, with a mean length of stay of 6 days. Prosthesis design was not associated with level of patient of patient satisfaction. The pre-operative joint specific score (OKS) was worse in those with a decreased level of satisfaction, in contrast the generic physical wellbeing score (SF-12 PCS) did not demonstrate a difference between groups (Table 1; Fig. 1). In addition the mental wellbeing (SF-12 MCS) was worse in those patients with a decreased level of satisfaction (Table 1; Fig. 1). Regression analysis demonstrated that the absence of renal disease or back pain, or a better pre-operative mental wellbeing (SF-12 MCS) were significant independent predictors of a patient perceived good to excellent level of satisfaction with their hospital stay (Table 2).

**Table 2:** Logistic regression analysis to identify independent pre-operative predictors of good to excellent satisfaction with hospital stay

Predictors in model	Odds ratio	95% CI		*p* value
		Lower	Upper	
Gender	1.09	0.82	1.37	0.57
Age	1.01	1.00	1.03	0.15
Comorbidity				
Heart disease	0.76	0.53	1.12	0.14
Hypertension	0.86	0.65	1.14	0.30
Lung disease	0.89	0.57	1.33	0.60
Vascular disease	0.71	0.38	1.33	0.28
Neurological disease	1.19	0.58	1.90	0.64
Diabetes mellitus	0.85	0.56	1.25	0.42
Gastric ulceration	1.40	0.66	2.16	0.38
Kidney disease	0.38	0.16	1.27	**0.03**
Liver disease	10.47	1.13	12.70	0.05
Anaemia	1.74	0.87	2.43	0.12
Back pain	0.75	0.56	1.04	**0.049**
Depression	0.80	0.53	1.21	0.28
Length of stay	0.99	0.94	1.03	0.56
Prosthesis				
PFC	Reference			
Triathlon	0.91	0.69	1.22	0.53
Kinemax	1.25	0.76	2.05	0.38
Functional measures				
OKS	1.01	0.99	1.04	0.27
SF-12 PCS	1.00	0.97	1.02	0.82
SF-12 MCS	1.03	1.02	1.04	<**0.001**

All variables (in Table 1) were all entered into the model using “enter” methodology (Nagelkerke *R* ^2^ = 0.06)

Significant values (p < 0.05) have been highlighted in bold

**Fig. 2:**
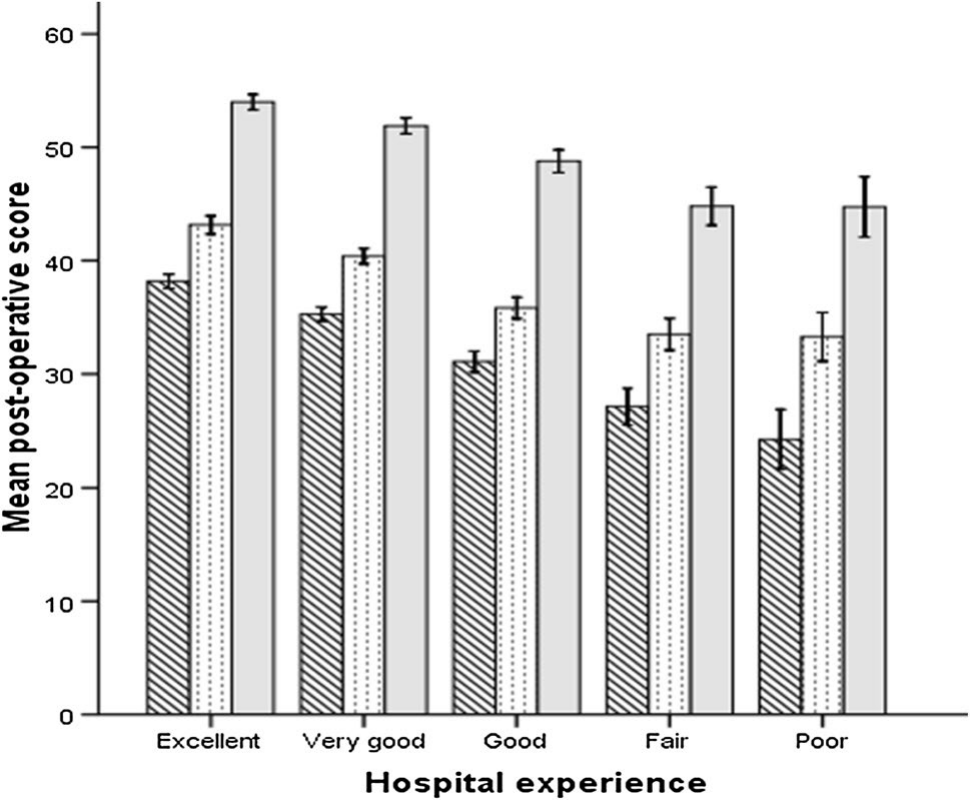
Post-operative OKS (*diagonal lines*), SF-12 PCS (*dots*) and MCS (*grey*) according to level of patient satisfaction with their hospital experience. 95% confidence interval *error bars*

**Fig. 3:**
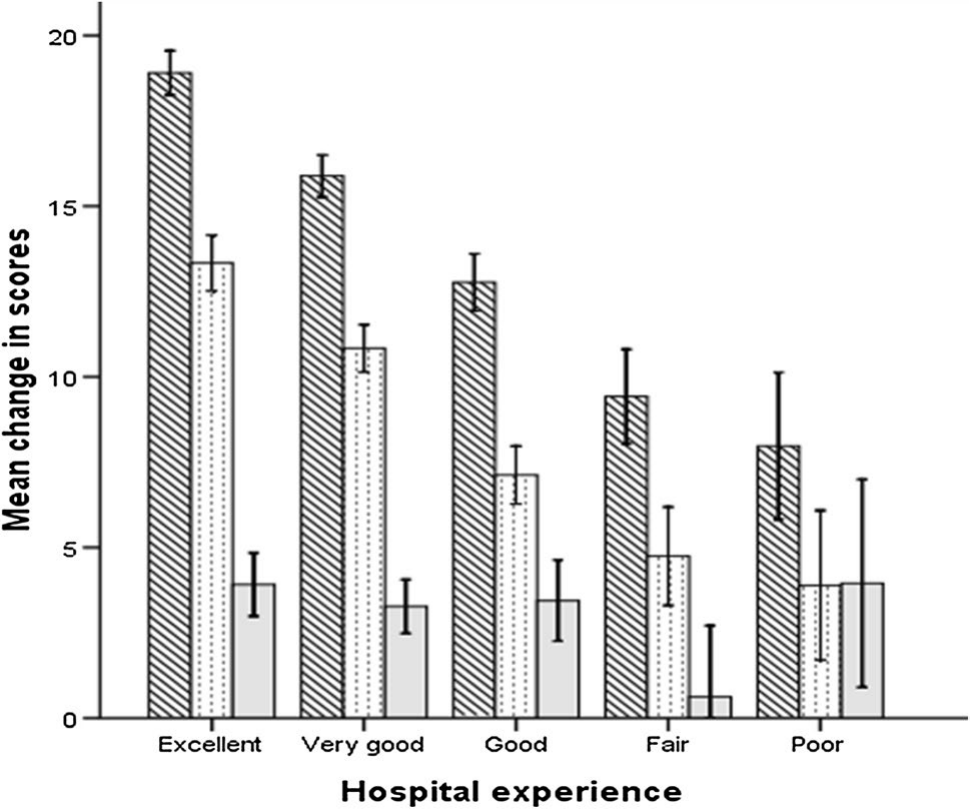
Improvement in OKS (*diagonal lines*), SF-12 PCS (*dots*) and MCS (*grey*) 1 year after TKA according to level of patient satisfaction with their hospital experience. 95% confidence interval *error bars*

Overall there was significant improvement in the OKS and the SF-12, for both the physical component score (PCS) and mental component score (MCS), 1 year after surgery for all patients (Table 3). However, the post-operative scores diminished significantly with decreasing level of satisfaction with hospital stay. There was a 14 point difference in the OKS, a 10 point difference in the SF-12 PCS, and a 9 point difference in the SF-12 MCS between those patients who rated their hospital stay as excellent compared to those who thought their stay was poor (Table 3; Fig. 2). However, all outcome measures improved significantly after TKA for all satisfaction groups, relative to pre-operative scores (Table 3). There was however a significant decrease in the improvement of the OKS and SF-12, both PCS and MCS with each decreasing level of satisfaction with hospital stay (Fig. 3).

**Table 3:** Post-operative outcome measures and the difference relative to pre-operative scores and satisfaction rate for the all patients and according to their level of satisfaction with hospital stay

Score	All patients (*n* = 2264)	Level of satisfaction with hospital stay					*p* value
		Excellent (*n* = 708)	Very good (*n* = 854)	Good (*n* = 429)	Fair (*n* = 184)	Poor (*n* = 89)	
OKS (SD)	34.3 (10.1)	38.1 (8.4)	35.3 (9.1)	31.1 (9.7)	27.2 (11.0)	24.3 (12.2)	<0.0001*
Difference (95% CI)	15.4 (15.0–15.7)	18.9 (18.2–19.5)	15.9 (15.3–16.5)	12.8 (11.9–13.6)	9.5 (8.2–10.9)	8.0 (5.8–10.1)	<0.0001*
*p* value**	<0.0001	<0.0001	<0.0001	<0.0001	<0.0001	<0.0001	
PCS (SD)	39.5 (10.7)	43.2 (10.8)	40.4 (9.9)	35.8 (9.9)	33.5 (9.5)	33.2 (10.1)	<0.0001*
Difference (95% CI)	10.1 (9.7–10.6)	13.3 (12.5–14.1)	10.9 (10.1–11.6)	7.1 (6.2–8.0)	4.9 (3.4 to 6.3)	3.8 (1.7–5.9)	<0.0001*
*p* value**	<0.0001	<0.0001	<0.0001	<0.0001	<0.0001	0.001	
MCS (SD)	51.1 (10.6)	53.9 (9.2)	51.9 (10.2)	48.8 (10.6)	44.8 (11.5)	44.8 (12.5)	<0.0001*
Difference (95% CI)	3.4 (2.9 –3.9)	3.9 (3.0–4.8)	3.4 (2.6 –4.1)	3.4 (2.3–4.6)	0.9 (−1.2–2.9)	3.8 (0.7–6.9)	0.06*
*p* value**	<0.0001	<0.0001	<0.0001	<0.0001	0.41	0.02	
Satisfied	1877 (84.8)	669 (94.5)	763 (89.4)	316 (74.0)	92 (50.3)	37 (42.0)	<0.001^†^
Unsatisfied (*n*, % of group)	382 (15.2)	39 (5.5)	90 (10.6)	111 (26.0)	91 (49.7)	51 (58.0)	

*ANOVA, **Paired *t* test, ^†^Chi square test

Multivariable linear regression analysis confirmed that a patient’s perception of their hospital experience was an independent predictor of change in their OKS, SF-12 PCS and MCS 1 year after TKA when adjusting for confounding variables (Table 4). There was a significant decrease in the improvement of the OKS, SF-12 PCS and MCS scores with each decreasing level of satisfaction relative to those patients who had an excellent experience. According to the regression models patients with an excellent hospital experience had a 10 point greater increase in the OKS and a 6 point greater increase in the SF-12 PCS and MCS at 1 year relative to those patients who had a poor experience.

**Table 4:** Multivariable linear regression analysis to identify independent predictors of change in OKS, SF-12 PCS and MCS 1 year after TKA

Model	Variable	*R* ^2^	*B*	95% CI		*p* value
				Lower	Upper	
Change in OKS	Excellent	0.38	Reference			
	Very good		−2.51	−3.30	−1.72	<0.001
	Good		−5.51	−6.48	−4.53	<0.001
	Fair		−8.92	−10.26	−7.57	<0.001
	Poor		−10.13	−11.98	−8.27	<0.001
Change in SF-12 PCS	Excellent	0.44	Reference			
	Very good		−2.13	−3.00	−1.72	<0.001
	Good		−4.93	−6.00	−3.97	<0.001
	Fair		−6.53	−8.00	−5.07	<0.001
	Poor		−5.92	−8.00	−3.84	<0.001
Change in SF-12 MCS	Excellent	0.59	Reference			
	Very good		−1.18	−2.02	−034	0.006
	Good		−3.21	−4.30	−2.13	<0.001
	Fair		−6.33	−7.79	4.86	<0.001
	Poor		−5.82	−7.81	−3.82	<0.001

All variables (in Table 1) were entered into each model using “enter” methodology

**Fig. 4:**
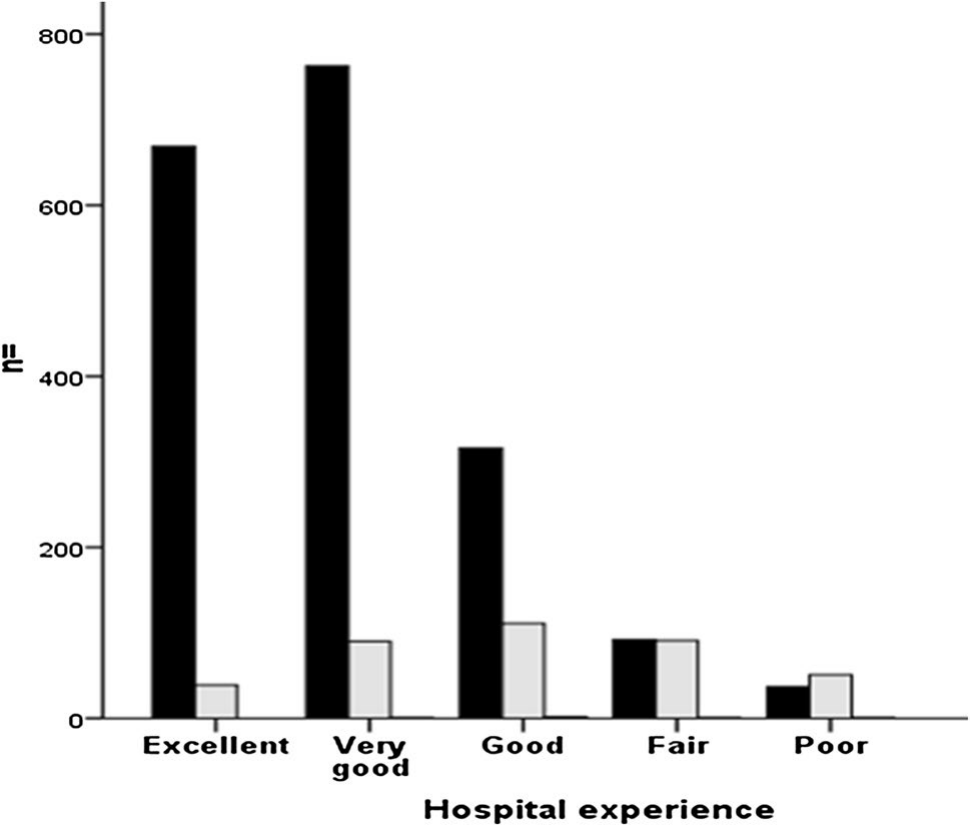
Number of patients satisfied (*black*) and not satisfied (*grey*) with their TKA 1 year after surgery according to level of patient satisfaction with their hospital experience

There were 1877 (83%) satisfied or very satisfied patients, with 267 (12%) being unsure, and 115 (5%) dissatisfied with their TKA. However, an additional 5 (0.2%) patients did not answer this question. The rate of satisfaction decreased from 96% in patients perceiving their hospital stay as excellent to 42% in patients perceiving their hospital stay as poor (Fig. 4). Logistic regression analysis was used to adjust for confounding variables between the groups, which confirmed that a patients perceived level of satisfaction with their hospital stay was a significant independent predictor of satisfaction with their TKA at 1 year (Table 5). Patients who perceived their hospital stay to be excellent were more than twice as likely to be satisfied with their TKA at 1 year compared to those patients rating their stay as very good, and nearly 20 times more likely than those rating their stay as fair or poor.

**Table 5:** Logistic regression analysis to identify the independent effect of perceived satisfaction of hospital stay on patient satisfaction with their TKA 1 year following surgery after adjusting for confounding variables

Level of satisfaction with hospital stay	*R* ^2^	Odds ratio	95% CI		*p* value
			Lower	Upper	
Excellent	0.39	Reference			
Very good		0.49	0.32	0.74	0.001
Good		0.16	0.10	0.24	<0.0001
Fair		0.06	0.04	0.10	<0.0001
Poor		0.05	0.02	0.09	<0.0001

All variables significant (in Table 1) were entered into the model using “enter” methodology

*Nagelkerke

**Fig. 5:**
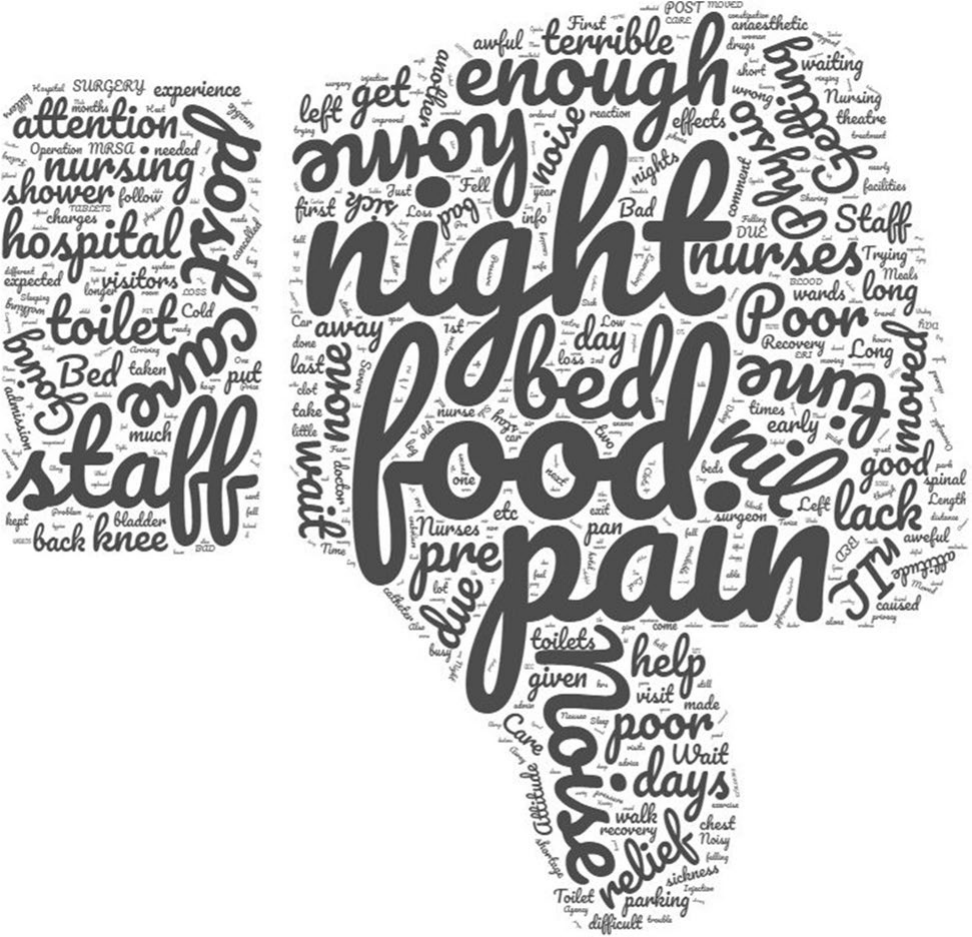
A word cloud illustrating the declared worst aspects of the patients’ hospital stay for the study cohort. The larger the word the more frequent patients used this to describe their stay

The ten most cited reasons for the worst aspect of hospital stay were: food (*n* = 585), pain (*n* = 107), night (*n* = 67), bed (*n* = 50), staff (*n* = 41), noise (*n* = 39), care (*n* = 38), going home to soon (*n* = 27), and toilet (*n* = 26). In total there were 903 words used to describe the worst aspect of their hospital stay which were cited 3201 times (Fig. 5). More than 70% of patients who perceived their hospital stay as fair or poor declared that the worst aspect of their stay was due to the food, staff, or environment (Table 6). Interestingly 27 patients, who defined their hospital stay as fair or poor, did so because the worse aspect was incurring “complications”, a retrospective sub group analysis was conducted on this cohort. Only 18 (67%) of the 27 patients had a documented complication (renal failure *n* = 2, wound leakage/dehiscence *n* = 5, deep vein thrombosis/pulmonary embolism *n* = 5, respiratory or urine infection *n* = 4, catheter for acute retention of urine *n* = 2). The remaining 9 patients were contacted and asked why they thought they had incurred a complication, the main issue was in relation to pain control with associated nausea and vomiting.

**Table 6:** Reasons why patients perceived their hospital stay as fair of poor

Worst aspect of hospital stay (*n*, % of group)	Fair (*n* = 184)	Poor (*n* = 89)
Food	45 (24.5)	15 (16.8)
Staff/care	36 (19.6)	26 (29.2)
Environment	48 (26.1)	22 (24.7)
Pain	14 (7.6)	7 (7.9)
Complication	16 (8.7)	11 (12.4)
Multiple of above	22 (12.0)	7 (7.9)
Other	3 (1.6)	1 (1.1)

## Discussion

This study has shown that a patient’s perception of their hospital stay affects the outcome of TKA. Patients with renal disease, back pain or worse mental wellbeing were more likely to be dissatisfied with their hospital stay. Decreasing level of satisfaction with hospital stay was associated with a significantly worse improvement in the OKS, SF-12 PCS and MCS, after adjusting for confounding factors. Patient satisfaction with their TKA was also significantly influenced by their hospital experience when adjusting for confounding factors, decreasing from 96% in those with an excellent experience to 42% in those with a poor experience.

A major limitation of our study was assessing patient satisfaction with their hospital care 6 months after surgery, which may be affected by recall bias. Other authors assessing satisfaction with hospital stay have assessed this approximately 1 month after surgery [2, 3]. A second limitation of the study was using a non-validated assessment tool to assess comorbidity, we simply recorded whether a specific comorbidity was present or not. A recent study using the validated Charlson comorbidity index demonstrated a worse functional outcome with increasing severity of the score [12]. However, we did include specific comorbidities in the regression models that have been shown to influence functional outcome and patient satisfaction, such as depression [7], back pain [10], general health [5], and diabetes [8], and adjusted for the effect of these upon outcome.

Prior studies analysing patient satisfaction with hospital stay, across all medical and surgical specialities, have demonstrated an 80% satisfaction rate with inpatient hospital stay [18, 23]. This is similar to our 88% rate of good to excellent level of satisfaction with inpatient stay after TKA. Increasing age [14] and smaller hospitals [24] have been shown to be associated with a greater level of patient satisfaction with their inpatient hospital experience. To our knowledge only a single study has assessed the effect of pre-operative patient demographics and level of function (SF-36) as predictors of satisfaction with hospital care after total hip replacement and TKA [4]. They demonstrated that pre-operative bodily pain and social functioning influenced patient satisfaction with care. We have shown using a larger cohort, after adjusting for confounding variables that renal disease, back pain and mental wellbeing are independent predictors of patient satisfaction with hospital stay after TKA. Such patients may benefit from expectation modification, through education [17], which may result in an improvement in their satisfaction with their hospital stay.

Baumann et al. [3] demonstrated that patients satisfied with their hospital stay after TKA resulted in an improved post-operative generic functional outcome (SF-36) score. We have affirmed the findings of Baumann et al. [3], finding an approximate two point decrease in the improvement of the generic SF-12 PCS and MCS for each drop in level of satisfaction with hospital stay. Furthermore, we have shown that hospital experience is an independent predictor of change in the OKS after TKA, which is an original observation. The improvement in the OKS decreased by approximately 2.5 points for each drop in level of satisfaction with hospital stay after adjusting for confounding factors. The minimal clinically important difference in the OKS and SF-12 score after TKA is thought to be between 4 and 5 points depending on satisfaction with pain and function [9]. Hence, the difference between two levels of satisfaction with hospital stay may be clinically important.

The demonstrated 85% rate of patient satisfaction with TKA at 1 year is consistent prior studies [16]. The novel aspect of our study was the significant decrease in the satisfaction rate with diminishing level of satisfaction with inpatient hospital experience. Age, medical and psychiatric comorbidity, pre-operative expectation and fulfilment of expectations, type of arthritis, and disease severity have all been shown to be determinants of patient satisfaction with TKA [16]. However, few of these are modifiable predictors of satisfaction. The effect of a patient’s perception of their hospital experience is a potential modifiable predictor of satisfaction of TKA. Hence, optimising a patient’s hospital experience by improving the quality of the food, care, and environment, being the most prevalent reasons of a fair or poor experience, may result in a significant increase in the rate of patient satisfaction with their TKA.

Patients with depression, back pain, and poor mental health have been shown to have a lower improvement in the OKS and SF-12 score, and a lower rate of satisfaction with their TKA [7, 10, 21]. Whether the perceived satisfaction with hospital stay is truly independent of such influencing factors needs to be affirmed in future studies, as we have shown patients with back pain and poor mental health are more likely to have a subjectively fair or poor hospital stay. However, when adjusting for these factors in the regression models it would seem that the effect of hospital stay is independent of such factors. Data from the National Joint Registry illustrated those patients undergoing TKA in an independent hospital or surgical treatment centre had a greater improvement in their OKS and EuroQol 5 dimension score relative to those patients who underwent a TKA in a National Health Service hospital [1]. Whether this reflects differing standards of care and hospital experience is not known, and should be assessed in future studies.

A patient’s perception of their inpatient hospital experience after their TKA is potentially an important modifiable predictor of functional outcome and satisfaction with their TKA 1 year after surgery. The reasons why patients perceive their hospital stay to be dissatisfactory after a TKA needs to be explored further in future studies and whether modification of such perceptions or improvement of the hospital environment result in a superior outcome.
